# Supplementary material for: Evaluation of the Antifungal Activities of Photorhabdus akhurstii and Its Secondary Metabolites against Phytopathogenic Colletotrichum gloeosporioides
Source: J Fungi (Basel). 2022 Apr 15;8(4):403. doi: 10.3390/jof8040403 (PMC9027565; doi:10.3390/jof8040403)
Supplement: Supplementary file 1 [file jof-08-00403-s001.zip › jof-1649917-supplementary.pdf]

Supplementary Information

Evaluation of the antifungal activities of *Photorhabdus akhurstii* and its secondary metabolites against phytopathogenic *Colletotrichum gloeosporioides*

Po-Wen Tu [1,2], Jie-Siang Chiu [3], Chih Lin [1], Chih-Cheng Chien [1], Feng-Chia Hsieh [4], Ming-Che Shih [1,3,*] and Yu-Liang Yang [1,2,*]

[1]Agricultural Biotechnology Research Center, Academia Sinica, Taipei 11529, Taiwan

[2]Biotechnology Center in Southern Taiwan, Academia Sinica, Tainan 71150, Taiwan

[3]Institute of Plant Biology, College of Life Science, National Taiwan University, Taipei 10617, Taiwan

[4]Biopesticide Division, Taiwan Agricultural Chemicals and Toxic Substances Research Institute, Council of Agriculture, Taichung, 41358, Taiwan

[*]Correspondence: ylyang@gate.sinica.edu.tw (Y.-L.Y.); mcshih@gate.sinica.edu.tw (M.-C.S.); Tel.: +886-6- 3032836 (Y.-L.Y.); +886-2-2787-2041 (M.-C.S.)

Tables

Table S1. The program performed on MPLC with the mobile-phase gradient

Table S2. The program performed on HPLC with the mobile-phase gradient

Table S3. NMR data of cepafungin I (Cep) and glidobactin A (Gli)

Table S4. Target genes and oligonucleotide primers sequences used in qRT-PCR

Table S5. Predicted BGCs of secondary metabolites from the genome sequences of *Photorhabdus akhurstii* sp. nov. 0813-124 phase I

Table S6. Comparison and predicted functions of encoded proteins in the glidobactin BGC for *Schlegelella brevitale*a sp. nov. DSM 7029 and *Photorhabdus akhurstii* sp. nov. 0813-124 phase I

Figures

Figure S1. MPLC profile of EA crude extract and concatenation portions.

Figure S2. ^{1}H-NMR spectrum of Cepafungin I.

Figure S3. ^{1}H- ^{1}H COSY NMR spectrum of Cepafungin I.

Figure S4. HSQC NMR spectrum of Cepafungin I.

Figure S5. ^{1}H-NMR spectrum of Glidobactin A.

Figure S6. ^{1}H- ^{1}H COSY NMR spectrum of Glidobactin A.

Figure S7. HSQC NMR spectrum of Glidobactin A.

Figure S8. Scheme of the separation PL1 extract and isolation of five bioactive peaks.

Figure S9. The LC-MS (EIC) profiles of active compounds in different incubation conditions.

Figure S10. Minimum inhibitory concentrations of PL1 extract and isolated compounds against *Colletotrichum gloeosporioides* spore.

References

Table S1. The program performed on MPLC with the mobile-phase gradient

Time (min)	Buffer A (%)	Buffer B (%)	Buffer C (%)	Flow rate (mL/min)
0-5	100	0	0	20
5-8	80	20	0	20
8-11	60	40	0	20
11-14	40	60	0	20
14-17	20	80	0	20
17-20	0	100	0	20
20-23	0	80	20	20
23-26	0	60	40	20
26-29	0	40	60	20
29-32	0	20	80	20
32-37	0	0	100	20

Table S2. The program performed on HPLC with the mobile-phase gradient

Time (min)	Buffer A (%)	Buffer B (%)	Flow rate (mL/min)
0	55	45	2.5
10	55	45	2.5
30	30	70	2.5
31	0	100	2.5
36	0	100	2.5
37	55	45	2.5
45	55	45	2.5

Table S3. NMR data of cepafungin I (Cep) and glidobactin A (Gli) [1]

Atom no.	Type	Stein et al., 2012 (500 MHz DMSO-d_6)		This study (600 MHz DMSO-d_6)		
		δ_H,mult. (*J* in Hz)	δ_C	δ_H,mult. (*J* in Hz)	δ_C	COSY
(Cep)1, 2	CH_3	0.84, d (3.25)	23.03	0.84, d (6.63)	22.29	(Cep)3
(Cep)3	CH	1.50, m	27.82	1.49, m	27.25	(Cep)1, 2, 4
(Cep)4	CH_2	1.15, m	38.86	1.15, m	38.15	(Cep)3, 5
(Gli)1	CH_3	0.86, t (7.0)	14.46	0.86, t (7.0)	14.23	(Gli)3
(Gli)3	CH_2	1.26, m	31.68	1.26, m	31.86	(Gli)1
(Gli)4	CH_2	1.26, m	26.21	1.26, m	26.23	
5	CH_2	1.26, m	27.08	1.26, m	26.25	
6	CH_2	1.26, m	29.21	1.26, m	28.28	7
7	CH_2	1.39, m	28.81	1.41, m	28.24	6, 8, 9
8	CH_2	2.13, m	32.76	2.13, m	32.20	7, 9, 10, 11
9	CH	6.11, m	142.68	6.10, m	142.12	
10	CH	6.19, m	129.49	6.20, m	128.38	
11	CH	7.00, dd (15.0, 10.0)	140.24	7.00, dd (15.12, 10.81)	139.60	10, 12
12	CH	6.13, m	123.49	6.12, m	123.18	
13	C(O)	–	165.88	–	165.31	
14	NH	7.91, d (9.0)	–	7.90, d (8.83)	–	15
15	CH	4.29, m	58.49	4.28, dd (8.81, 4.1)	58.04	14, 16
16	CH	3.97, m	67.18	3.95, m	66.78	15, 17, 34
17	CH_3	1.0, d (10)	20.46	1.00, d (6.31)	19.79	17
18	C(O)	–	169.86	–	169.13	
19	NH	7.76, d (7.0)	–	7.74, d (7.82)	–	20
20	CH	4.34, m	51.66	4.68, m	50.85	19, 30
21	C(O)	–	171.44	–	169.22	
22	NH	8.69	–	8.67, br	–	23
23	CH	4.37, m	45.21	4.37, m	44.45	22, 24, 26
24	CH_3	1.22, m	19.05	1.20, m	18.5	23
25	CH	6.41, d (15.0)	143.56	6.18, m	142.00	23, 26
26	CH	6.19, m	140.23	6.40, d (12.77)	143.05	23, 25
27	C(O)	–	168.09	–	169.17	
28	NH	7.44, t (5.5)	–	7.42, t (6.27)	–	29
29	CH_2	3.02, m	40.40	3.03, m	39.88	28, 30
30	CH_2	1.45, m	40.07	1.43, m	39.6	29, 31
31	CH	3.58, m	67.57	3.56, m	66.47	20, 30, 32, 33
32	CH_2	1.85, m/1.58, d (11.5)	42.93	1.84, m/1.58, d (10.41)	44.45	20, 31
33	OH	Unassigned	–	4.67, br	–	31
33	OH	Unassigned	–	4.87, br	–	16

[1] The ^{13}C chemical shift was assigned based on HSQC and HMBC.

Table S4. Target genes and oligonucleotide primers sequences used in qRT-PCR

Gene	Description	Blast hit		Primer sequence
		E-value	Accession	
09515	Nuclear transport factor 2 family protein	1E-82	WP_036807791.1	F: TTTACGACGCCCTTCATTTTCC
				R: TACGCAAACTGGTTTCATTGCT
09520	Non-ribosomal peptide synthetase (NRPS)	0	WP_046396347.1	F: GTCTGCTTGCCGAGTTGTTG
				R: CAACGTGATTTCAACGCCGA
09525	Major facilitator superfamily transporter	0	WP_046396348.1	F: TCGTCAGGGACAACCTCTCT
				R: ATGTCTGCCTGAGAACCACG
09530	Hybrid NRPS/type I polyketide synthase (PKS)	0	WP_052739462.1	F: CAATTTATGCCTCTGGCCGC
				R: ATGGCCCTTGCTTCTGTACC
09535	2OG-Fe dioxygenase family protein	0	WP_105395866.1	F: ATGATGCGGCGATTTGTCAC
				R: TCACTCTGTTCGAGTGGGGT

Table S5. Predicted BGCs of secondary metabolites from the genome sequences of *Photorhabdus akhurstii* sp. nov. 0813-124 phase I

Cluster	BGC-type	Most similar known cluster
1	Bacteriocin	-
2	Ppysks	-
3	Other	-
4	Terpene	Carotenoid BGC<br>(83% of genes show similarity)
5	NRPS	Rhabdopeptides BGC<br>(100% of genes show similarity)
6	NRPS	-
7	Thiopeptide	O-antigen BGC<br>(14% of genes show similarity)
8	T1PKS - NRPS	Luminmycin BGC<br>(100% of genes show similarity)<br>Glidobactin BGC<br>(26% of genes show similarity)
9	Arylpolyene	-
10	Resorcinol	Isopropylstilbene BGC<br>(100% of genes show similarity)
11	T1PKS - NRPS	Yersiniabactin BGC<br>(4% of genes show similarity)
12	NRPS	Streptomycin BGC<br>(2% of genes show similarity)
13	NRPS	Turnerbactin BGC<br>(23% of genes show similarity)
14	NRPS	Xenoamicins BGC<br>(12% of genes show similarity)
15	NRPS	Glidobactin BGC<br>(15% of genes show similarity)
16	NRPS	-
17	T1PKS	-
18	NRPS	Distamycin BGC<br>(14% of genes show similarity)
19	T1PKS - NRPS	-
20	T1PKS - NRPS	Xenocoumacin BGC<br>(78% of genes show similarity)
21	T2PKS	Anthraquinone BGC<br>(100% of genes show similarity)
22	Siderophore	Desferrioxamine_B BGC<br>(60% of genes show similarity)

Table S6. Comparison and predicted functions of encoded proteins in the glidobactin BGC for *Schlegelella brevitalea* sp. nov. DSM 7029 and *Photorhabdus akhurstii* sp. nov. 0813-124 phase I [2,3]

S. brevitalea		***P. akhurstii***		
**Gene (Product size)**	**Encoded putative function**	**Gene (Product size)**	**Identity (%)**	**Similarity (%)**
glbA (338 aa)	Transcriptional regulator	-	-	-
glbB (287 aa)	Lysine 4-hydroxylase	*09535* (272 aa)	49.0	62.4
glbC (4182 aa)	NRPS/PKS	*09530* (4129 aa)	51.6	66.3
glbD (437 aa)	Transporter	*09525* (421aa)	49.3	64.8
glbE (74 aa)	MbtH-like protein	-	-	-
glbF (1084 aa)	NRPS	*09520* (1066 aa)	49.4	64.8
glbG (122 aa)	Nuclear transport factor 2 family protein	*09515* (119 aa)	50.0	60.7
glbH (473 aa)	2-nitropropane dioxygenase	-	-	-

[1] aa, amino acid; NRPS, non-ribosomal peptide synthetase; PKS, polyketide synthase.

[2] Identities and similarities in amino acid sequences of corresponding proteins were calculated using the online platform EMBOSS needle [4].

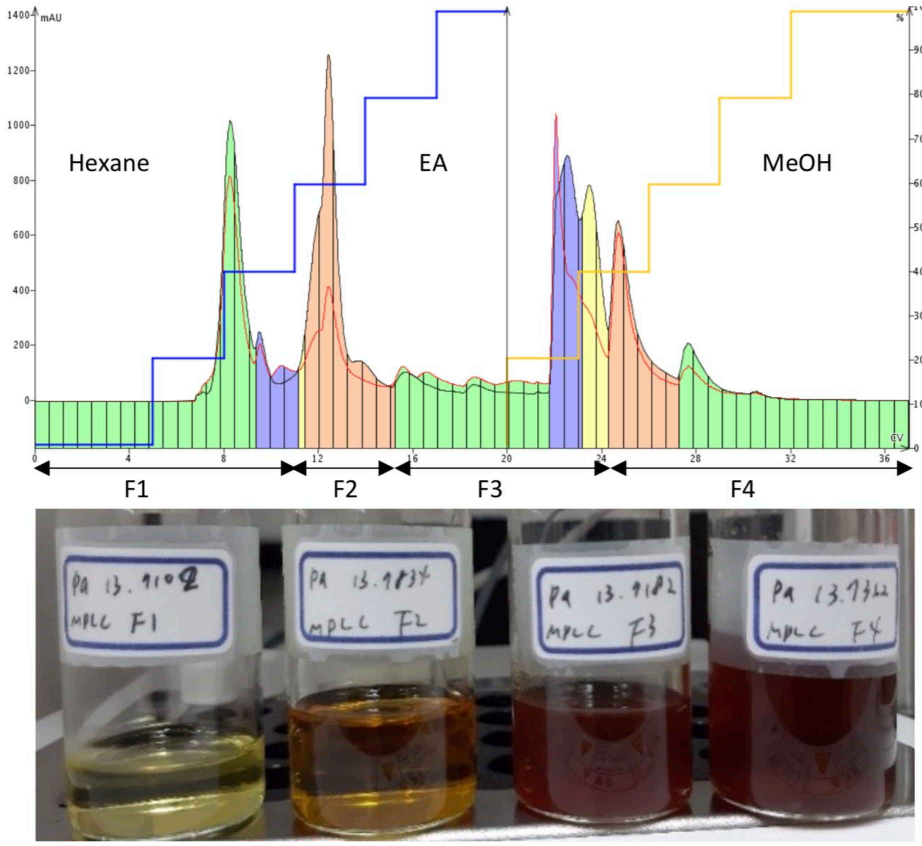

Figure S1. MPLC profile of EA crude extract and concatenation portions.

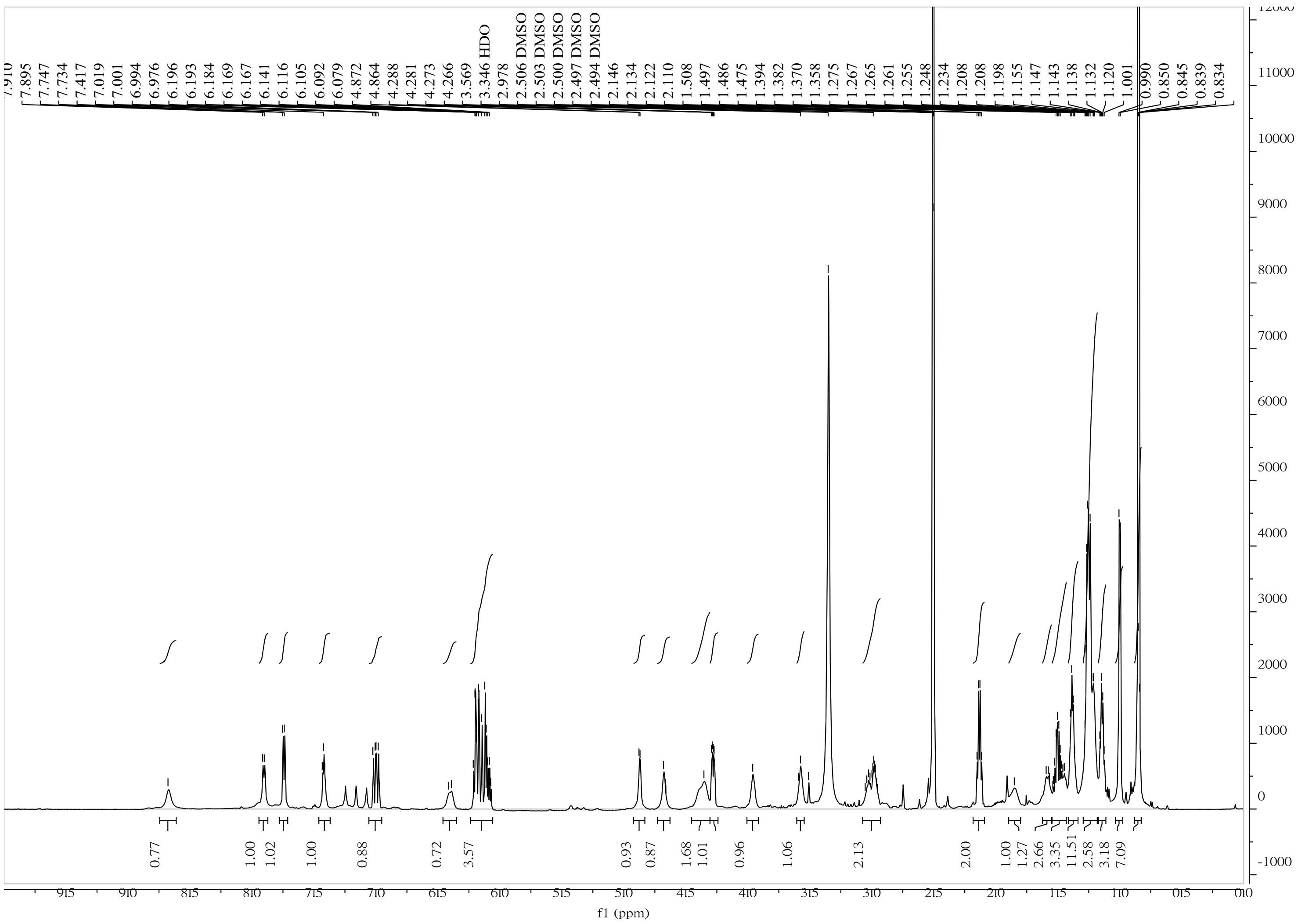

Figure S2. ^{1}H-NMR spectrum of Cepafungin I.

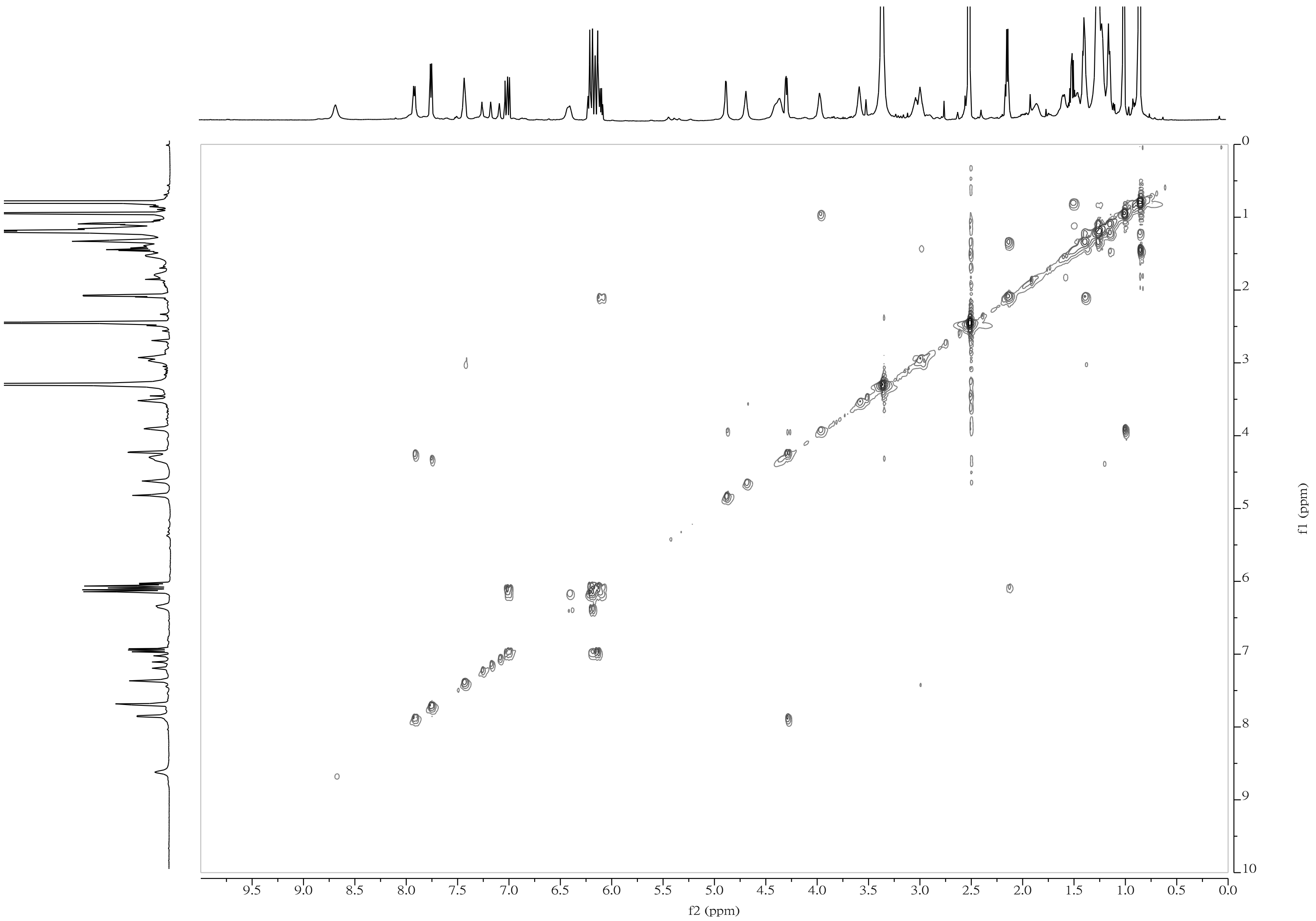

Figure S3. ^{1}H-^{1}H COSY NMR spectrum of Cepafungin I.

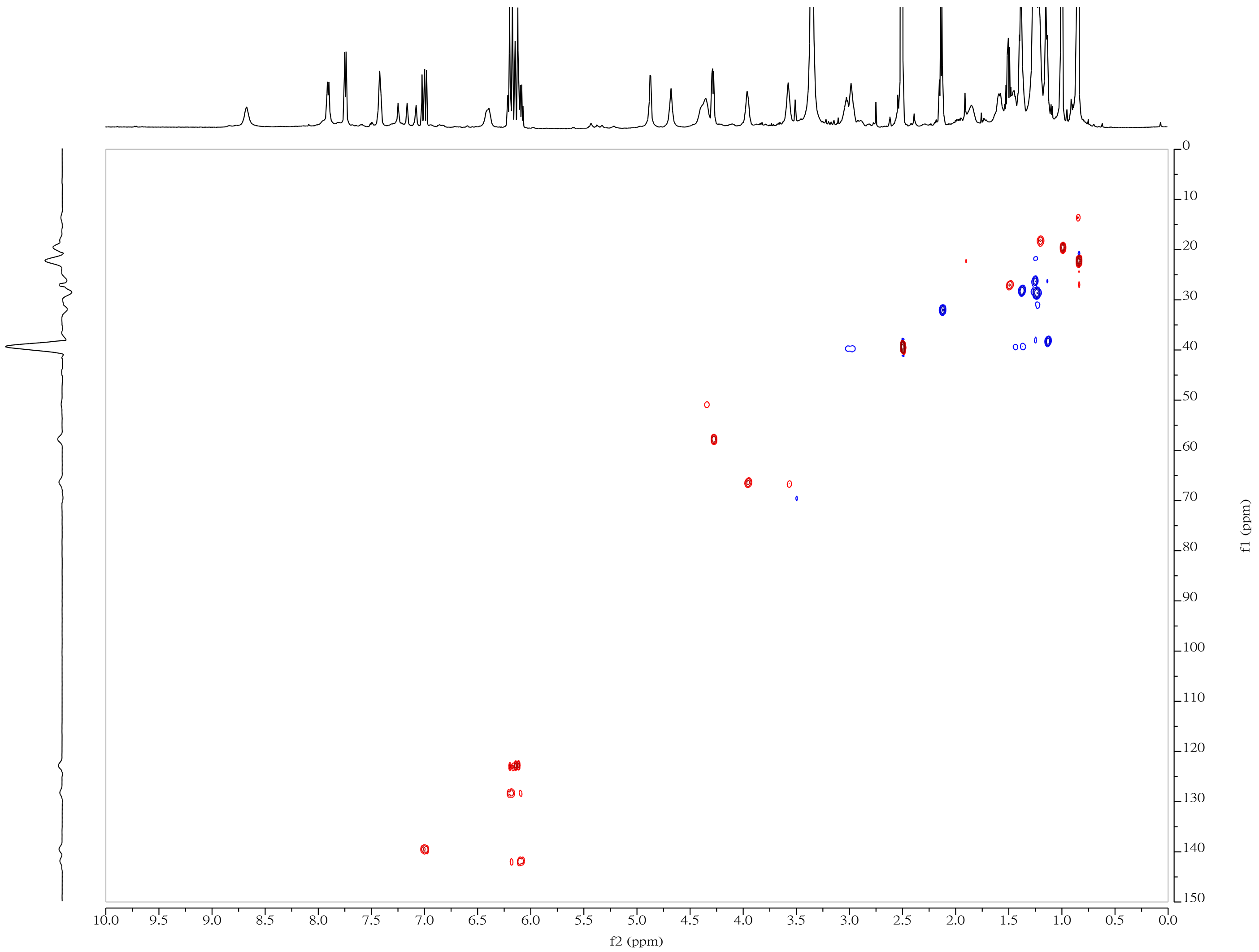

Figure S4. HSQC NMR spectrum of Cepafungin I.

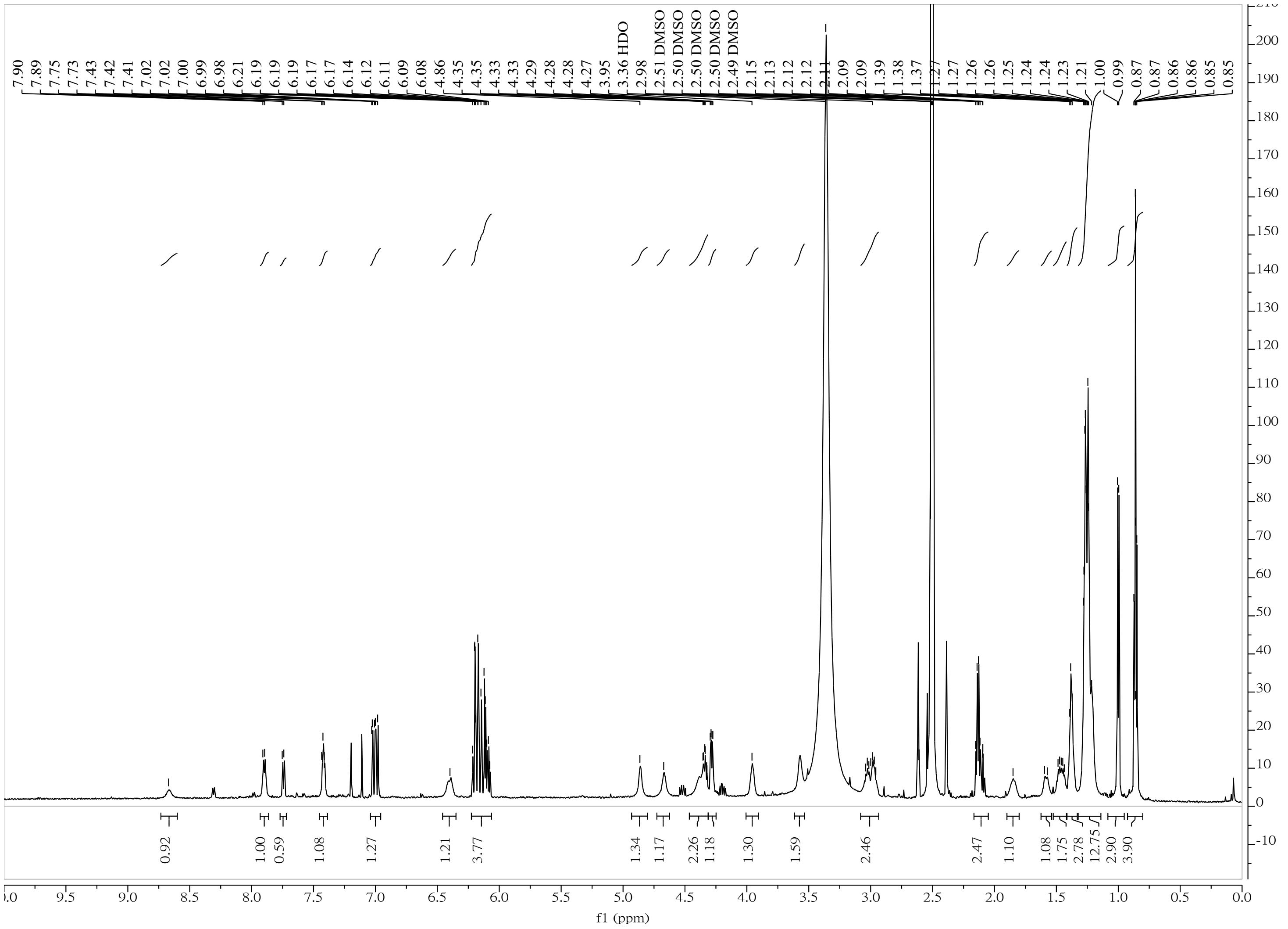

Figure S5. ¹H-NMR spectrum of Glidobactin A.

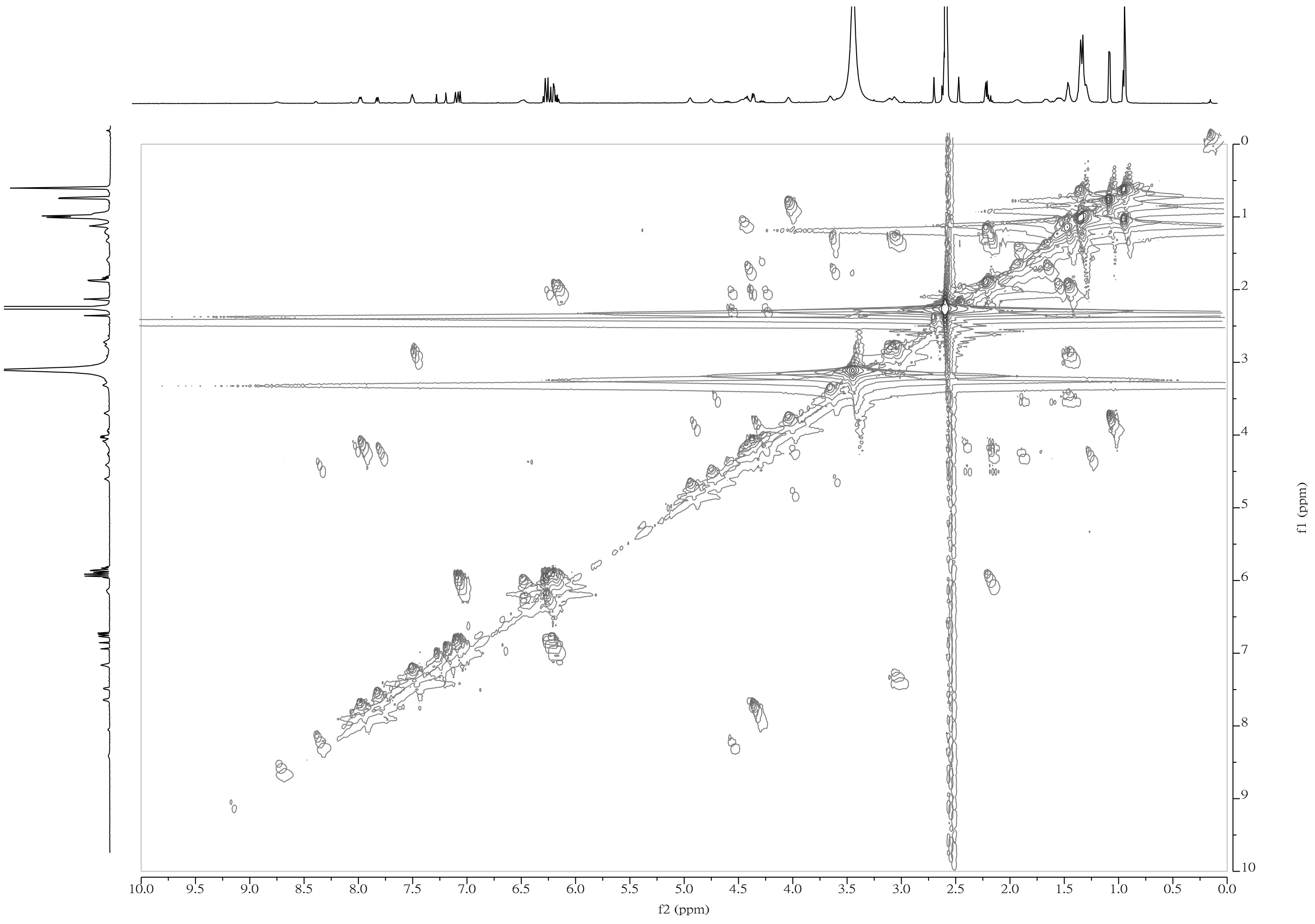

Figure S6. ^{1}H- ^{1}H COSY NMR spectrum of Glidobactin A.

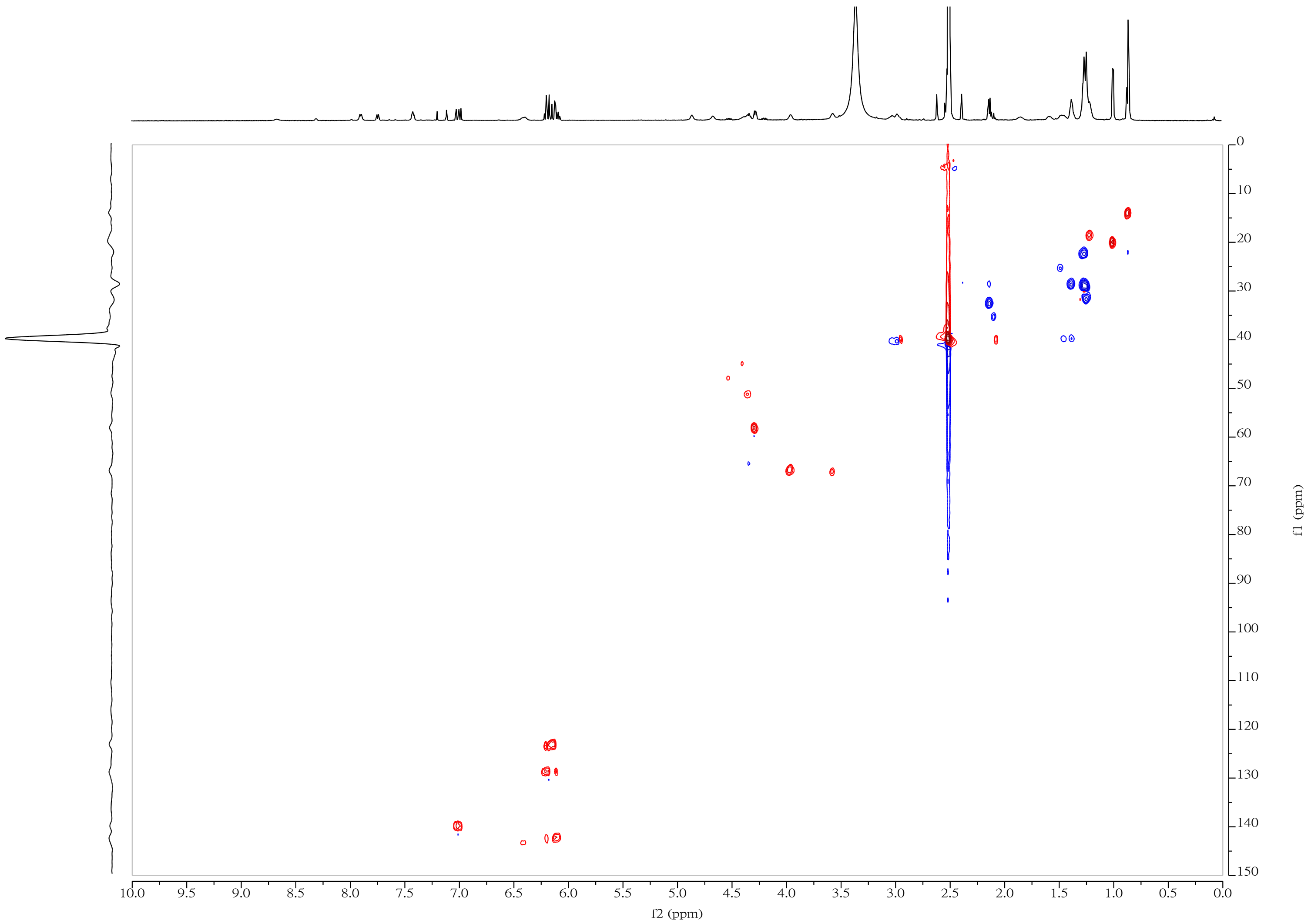

Figure S7. HSQC NMR spectrum of Glidobactin A.

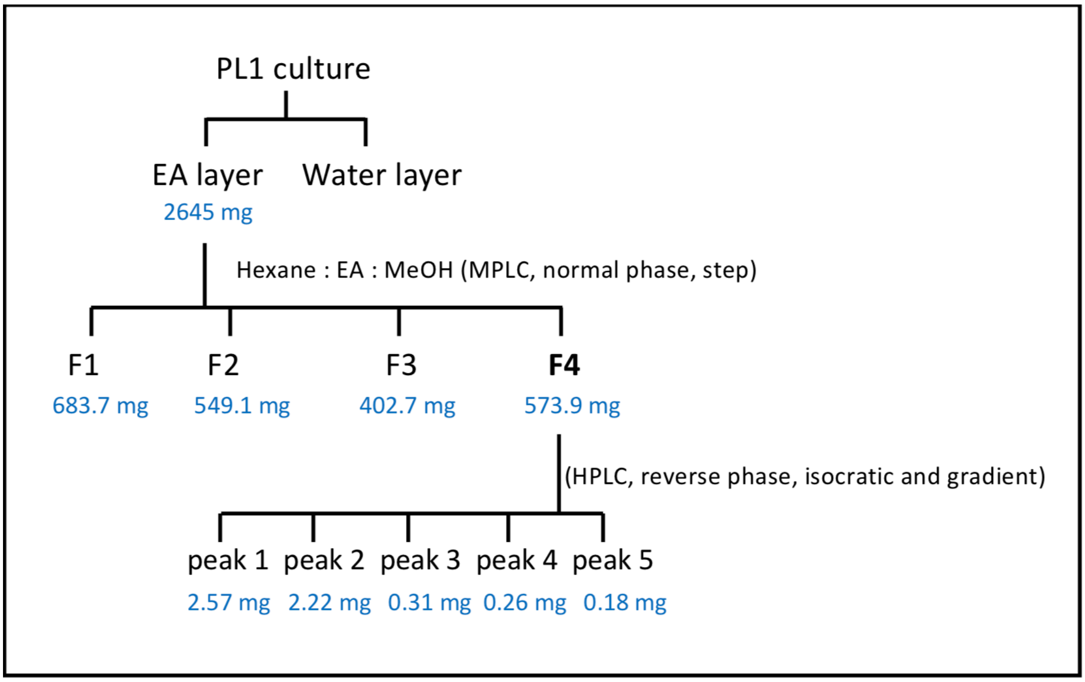

Figure S8. Scheme of the separation PL1 extract and isolation of five bioactive peaks.

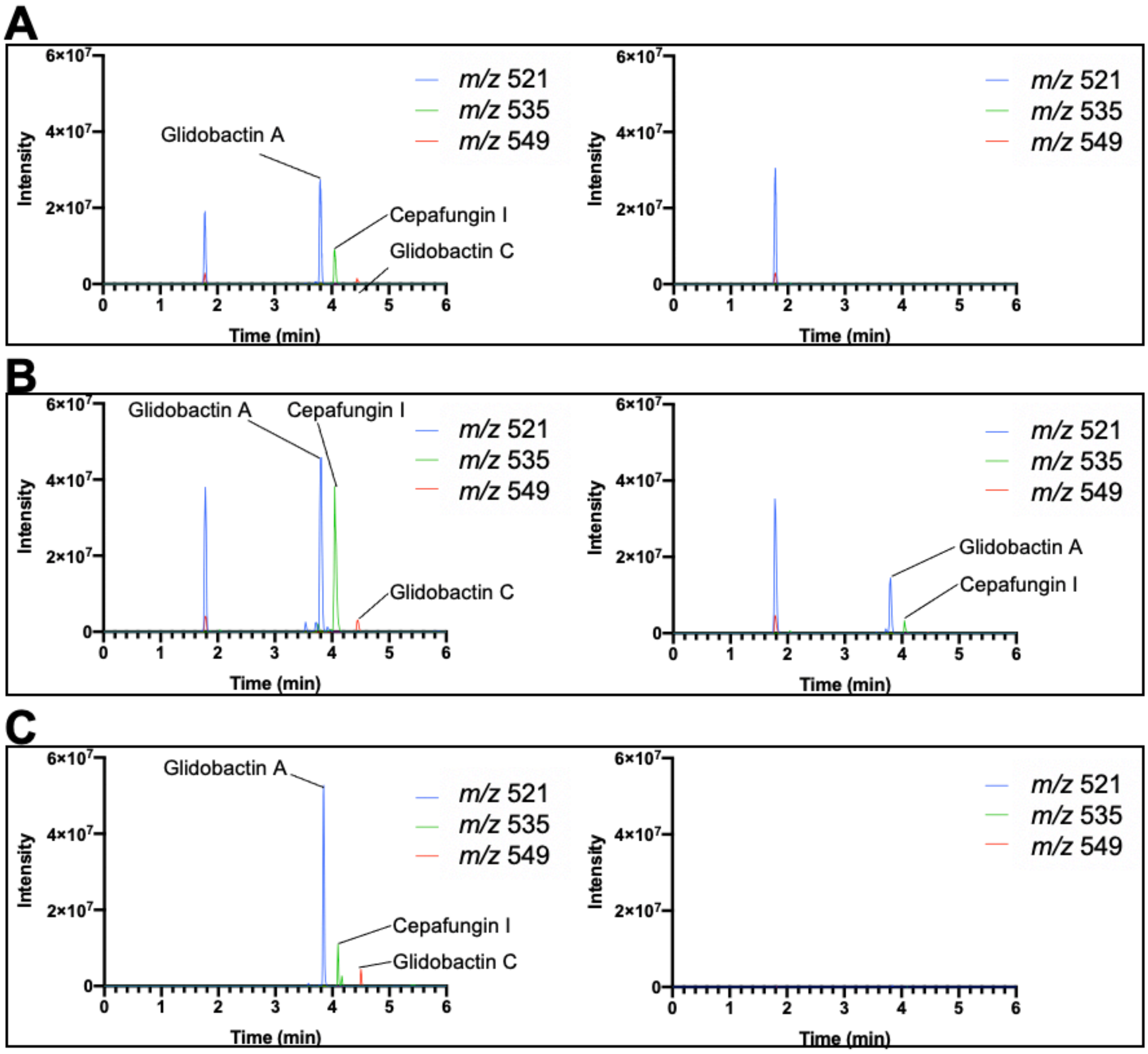

Figure S9. The LC-MS (EIC) profiles of active compounds in different incubation conditions, including LP agar (**A**), PDA (**B**), and PDB containing 0.5% tryptone (**C**) between PL1 (left) and PL2 (right).

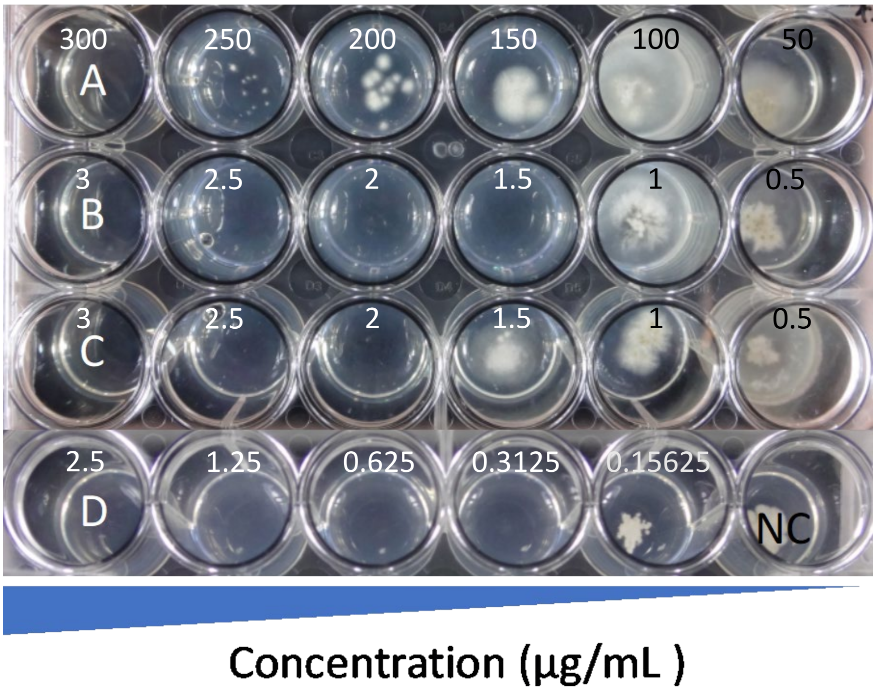

Figure S10. Minimum inhibitory concentrations of PL1 extract and isolated compounds against *Colletotrichum gloeosporioides* spore. **A**: PL1 crude extract; **B**: glidobactin A; **C**: cepafungin I; **D**: carbendazim. MICs were determined with consistent results repeated three times. NC: negative control.

References

1. Stein, M.L.; Beck, P.; Kaiser, M.; Dudler, R.; Becker, C.F.W.; Groll, M. One-shot NMR analysis of microbial secretions identifies highly potent proteasome inhibitor. *Proc. Natl. Acad. Sci. U.S.A.* **2012**, 109, 18367–18371, doi:10.1073/pnas.1211423109.
2. Zhao, L.; Le Chapelain, C.; Brachmann, A.O.; Kaiser, M.; Groll, M.; Bode, H.B. Activation, structure, biosynthesis and bioactivity of glidobactin-like proteasome inhibitors from *Photorhabdus laumondii*. *Chembiochem* **2021**, *22*, 1582–1588, doi:10.1002/cbic.202100014.
3. Bian, X.; Plaza, A.; Zhang, Y.; Müller, R. Luminmycins A–C, cryptic natural products from *Photorhabdus luminescens* identified by heterologous expression in *Escherichia coli*. *J. Nat. Prod.* **2012**, *75*, 1652–1655, doi:10.1021/np300444e.
4. Madeira, F.; Park, Y.M.; Lee, J.; Buso, N.; Gur, T.; Madhusoodanan, N.; Basutkar, P.; Tivey, A.R.N.; Potter, S.C.; Finn, R.D.; et al. The EMBL-EBI search and sequence analysis tools APIs in 2019. *Nucleic Acids Res.* **2019**, *47*, W636–W641, doi:10.1093/nar/gkz268.
